# Development of a Hydrocortisone Orodispersible Thin Film Containing Its Succinate Prodrug

**DOI:** 10.3390/ph18010086

**Published:** 2025-01-13

**Authors:** Clément Boisseillier, Lucas Demange-Labriet, Dulanjalee Kariyawasam, Pauline Marchadour, Anne-Sophie Fauqueur, Maxime Annereau, Lucas Denis, Camille Cotteret, Salvatore Cisternino, Arnaud Schweitzer-Chaput

**Affiliations:** 1 Service Pharmacie, Hôpital Universitaire Necker-Enfants Malades, Assistance Publique des Hôpitaux de Paris (AP-HP), 149 Rue de Sèvres, F-75015 Paris, France; clement.boisseillier@aphp.fr (C.B.); lucas.demangelabriet@aphp.fr (L.D.-L.); anne-sophie.fauqueur@aphp.fr (A.-S.F.); camille.cotteret@aphp.fr (C.C.); arnaud.schweitzerchaput@aphp.fr (A.S.-C.); 2 Service d’Endocrinologie, Diabétologie, Gynécologie Pédiatriques, Hôpital Universitaire Necker-Enfants Malades, Assistance Publique des Hôpitaux de Paris (AP-HP), 149 Rue de Sèvres, F-75015 Paris, France; dulanjalee.kariyawasam@aphp.fr; 3 Institut Cochin, Inserm U1016, Université Paris Cité, F-75014 Paris, France; 4 Institut Imagine, Inserm U1163, F-75015 Paris, France; 5 Service Pharmacie, Institut Gustave Roussy, F-94800 Villejuif, France; maxime.annereau@gustaveroussy.fr (M.A.); lucas.denis@gustaveroussy.fr (L.D.); 6 Inserm UMRS 1144, Université Paris Cité, 4, Avenue de l’Observatoire, F-75006 Paris, France

**Keywords:** adrenal hyperplasia, drug compounding, hydrocortisone, 21-hydroxylase deficiency, pediatrics, personalized medicine, pharmacy service

## Abstract

Orodispersible thin film (ODF) is an innovative dosage form that allows for adjustable dosing and improved patient compliance. It is administered by mouth, where it dissolves, making it suitable for children. Objectives: The aim of the study was to develop and characterize an optimal ODF formulation containing equivalent hydrocortisone at 0.5 mg/cm^2^ using the solvent-casting method. A stability-indicating assay for the simultaneous quantification of hydrocortisone and hydrocortisone 21-hemissucinate (HMS) was developed. ODFs were characterized by organoleptic properties and by testing for uniformity of mass, content, stability, thickness, and dissolution. Results: When optimized, ODF is thin, flexible, and transparent, making it suitable for production in hospital pharmacies using standard equipment. In contrast to the water-insoluble hydrocortisone, the HMS-loaded cast gel successfully satisfied the tests, including content uniformity. Disintegration appeared acceptable as compared to the commercial grade ondansetron ODF (Setofilm^®^). The physicochemical stability of the active ingredients (i.e., HMS, hydrocortisone) contained in the ODF at 0.5 mg/cm^2^ is demonstrated for at least 84 days at 23 °C. Conclusion: The ODF formulated with the water-soluble hydrocortisone prodrug HMS allows accurate drug level to be achieved, thus opening up new opportunities for use in pediatric patients.

## 1. Introduction

Hydrocortisone (HCT) is a glucocorticoid that is mainly used as a replacement therapy for children with adrenal insufficiency and also in a variety of autoimmune and inflammatory diseases. Adrenal insufficiency is a group of autosomal recessive genetic disorders that result in a total or partial deficiency of an enzyme involved in adrenal steroidogenesis. A mutation found in over 95% of cases results in a congenital deficiency of 21-hydroxylase, which at least impairs cortisol biosynthesis [1,2]. Hormone replacement therapy with glucocorticoids and/or mineralocorticoids is the standard of care. To mimic the physiological hormonal peak, an HCT formulation with immediate drug release is administered in three doses per day at 10 to 15 mg/m^2^/day, corresponding to ~1 to 2 mg per dose for a child [3]. In addition, dose adjustment is regularly necessary due to growth of the child. Therefore, a dosage form allowing for safe dose adjustment is necessary. Only one licensed HCT treatment, in the form of granules for oral suspension, has been developed for children under the trade name Alkindy^®^ (Diurnal, Cardiff, United Kingdom) [4]. However, this treatment is unavailable in many countries, and the only approved oral products containing HCT are only suitable to be used in the adult population. Consequently, dose adjustments are required for the pediatric population, which could be achieved by crushing adult tablets and suspending them extemporaneously in liquid by the family or caregiver. However, this practice is associated with a high risk of inaccurate dosage [5]. In this context, a safer alternative is the production of hard capsules, which are prepared mainly by hospitals or community pharmacies. However, the quality of these HCT pharmaceutical preparations is highly variable, which can have important consequences in the therapeutic management [6].

There is a growing interest in orodispersible thin film (ODF) formulations, as they allow for better compliance in populations with swallowing difficulties, such as elderly patients and children [7]. Indeed, recent studies have shown an improvement in the acceptability and palatability of ODFs in young children in comparison with oral liquid formulations [7,8]. The European Pharmacopeia provides the following definition for ODFs: “single- or multilayer sheets of suitable materials, to be placed in the mouth where they disperse rapidly” [9]. ODFs are designed to dissolve directly in the oral cavity, allowing the incorporated active pharmaceutical ingredient (API) to be swallowed with saliva. However, ODFs also have their limitations, particularly in terms of production and the need for a homogeneous and reproducible film [10].

While the production of ODFs can be automated, it can also be performed using a manual process, making it feasible for production in hospital or community pharmacies. Recently, various methods for ODF production have been explored, including 3D printing and solvent-casting. The most suitable method for community or hospital pharmacies is solvent-casting, due to its ease of implementation and significantly lower cost compared to 3D printing [11].

Briefly, to produce an ODF, the API and excipients are dissolved in water to form a gel, which is then poured and dried to form a large film which is cut into individual units of the desired size and dose. The main components of an ODF include the API, a polymeric agent such as maltodextrin or cellulose derivatives, a plasticizer such as glycerol, and other excipients to improve disintegration and mouth feel [12,13]. The low water content of ODFs provides superior microbiological and chemical stability comparable to that of solid oral dosage forms [14]. In addition, the pharmaceutical preparation process ensures uniform distribution of the API, the large-cast gel can be cut into multiple fragments whose surface area corresponds to the dose to be administered, enabling pharmacists to pre-prepare customized doses tailored to individual patient needs [15].

One of the major advantages of ODFs is their ability to disintegrate quickly. Among the parameters that influence disintegration, the solubility of the API plays a crucial role in the properties of ODFs. In particular, the water solubility of the API promotes the disintegration process, as well as ensuring uniformity and that each individual ODF unit will deliver the intended dose [11]. A water-insoluble API must be homogeneously and stably dissolved in the casting solution to ensure uniformity of the ODF API content. The low solubility of HCT in water is a critical issue when formulating ODF with HCT. Therefore, several strategies are possible, such as the use of lipid complexes or cyclodextrins to make HCT more water-soluble. However, an intravenous water-soluble form of HCT, hydrocortisone hemisuccinate (HMS), was chosen for our study. This ester HCT prodrug also has the advantage of releasing HCT by spontaneous hydrolysis.

The aim of this study is to develop an appropriate casting solution and production method for producing ODFs with a reproducible dose of HCT/HMS. A variety of excipients and conditions, with or without the incorporation of HMS into the casting gel, will be investigated to achieve this goal.

## 2. Results

### 2.1. Stability-Indicating Liquid Chromatography HMS and HCT Assay

#### 2.1.1. Validation of the HPLC Assay Method

The HMS calibration curve was linear between 20 µg/mL and 80 µg/mL (Table S1) (y = 1.442 (± 0.019) x + 1.196 (± 1.062); r^2^ = 0.998). The HMS retention time was ~20.5 min. Selectivity was obtained, and no matrix effect was demonstrated. As shown in Table S2, the HMS assay precision was ≤4%, the accuracy was not less than 95% for all HMS concentrations tested, and the repeatability was <3% (n = 9). The HMS LOD and LOQ were 2.4 µg/mL and 7.4 µg/mL, respectively, which was acceptable for the concentration range measured.

The HCT calibration curve was linear between 0.50 µg/mL and 50 µg/mL (Table S3) (y = 2.132 (± 0.004) x + 0.164 (± 0.086); r^2^ = 0.999). The HCT retention time was ~9.5 min. Selectivity was assessed—there was no peak deformation or change in the UV spectra in the presence of excipients, and no matrix effect was demonstrated. As shown in Table S4, the HCT assay precision was ≤2%, the accuracy was not less than 95% for all HCT concentrations tested, and the repeatability was <3% (n = 9). The HCT LOD and LOQ were 0.13 µg/mL and 0.40 µg/mL, respectively, which was acceptable for the concentration range measured.

#### 2.1.2. Selectivity of the HMS and HCT Analytical Method

The typical chromatogram of HMS and HCT are shown in Figure S1. The HCT chromatogram shows one peak with retention times of ~9 min. The HMS chromatogram shows three peaks with retention times of ~9.2 min (~2% of total area; HCT), ~11 min (~1% of total area; peak 2), and ~20.5 min (97% of total area; HMS). The chromatogram peak named “peak 2”, observed at ~11 min, remains an unidentified HMS Upjohn^®^ impurity (Figure S1). The presence of traces of HCT in this HMS solution prepared extemporaneously is also indicated by this analysis.

HMS is particularly sensitive under acidic and alkaline conditions, as shown by forced degradation studies (Table S5). Under these conditions, the degradation of HMS is associated with the appearance of HCT in an inversely proportional manner (Figure S2). HCT is also sensitive to extreme pH conditions but to a lesser extent than HMS (Table S5). In those conditions, degradation peaks with similar retention times regarding the two molecules were found.

Using DAD, no degradation products were found to be co-eluted with HCT or HMS peaks. Furthermore, no peak deformation or modification of the UV spectra of HCT or HMS was observed (Figure S3). Thus, the developed method indicates good specificity for studying HMS and HCT degradation and allows for its use as a stability-indicating method.

### 2.2. ODF Formulation Studies

#### 2.2.1. Preparation of the Casting Gel and ODFs

Several conditions and parameters were studied to optimize ODFs during all four preparation steps:

(1) Preparation of the casting gel: to obtain a perfectly homogenous gel, all the components except HPC and HMS were mixed in water. Once perfectly solubilized, the solution was heated to 40 °C, as HPC is insoluble over 38 °C. This temperature avoided the formation of small gelatinous masses and therefore improved the dispersion of the HPC. These steps were mandatory to avoid clots and aggregates in the casting gel, resulting in an ODF that would break at the slightest contact. To limit the exposure of the API to heating process, it was incorporated just before the HPC. The solution was then brought to room temperature under stirring, allowing the gel to form gradually.
(2) Gel formation and degassing: The solution had to be left to stand for several hours (~10 h) until the HPC was completely dispersed. It was ready for spreading when the gel was visually completely homogeneous and the air bubbles were completely removed. The absence of air bubbles was essential for obtaining a complete ODF with a reproducible and accurate API content. Degassing at +4 °C showed no effect versus ambient temperature as it took several hours to remove all the trapped air. Use of an ultrasonic bath for 5 min helped to raise air bubbles in the upper half of the recipient, saving a few hours in this stage.
(3) Casting process: the use of greaseproof paper was not satisfactory, as it absorbed too much water, resulting in an uneven thickness and discontinuities in the film. In addition, it did not allow for a good drying. A glass plate allowed for the formation of a smooth film, but demolding was impossible. Polyolefin liner allowed for the formation of a smooth film with very easy demolding, but we did not manage to stretch the liner enough, resulting in irregularities in the thickness of the film. Food grade silicone was the best tested surface, giving a smooth film that was easy to demold. This surface has been selected for the rest of study.

#### 2.2.2. ODF Formula Optimization

API: HMS was chosen as the API because of its better water solubility (~100 mg/mL in water), which enables an homogeneous aspect of the gel to be obtained at a difference of HCT (~0.3 mg/mL). Indeed, the use of HCT did not result in a homogeneous gel, and ODFs obtained after drying were extremely brittle.

Surfactant (glyceryl monolinoleate/tween 80; 75%/25%): without a surfactant in the formulation, the film showed significant shrinkage at one or two corners of the film area during drying on silicone. At 0.1%, some holes started to appear in the film. Satisfactory results were obtained at 0.15% and 0.2%, with no shrinkage and acceptable elasticity. Above 0.2%, the elasticity increased and became suboptimal, making it difficult or impossible to manipulate the film.

HPC and povidone K25: 5% povidone formulations were considered optimal, as the other percentages tested (i.e., 7.5 and 10%) resulted in a non-flexible ODF with a “plastic” appearance even with a lower percentage of HPC (3%).

Organoleptic tests (e.g., tactile, visual) of the casting gel containing 4% to 6% HPC showed that the gel could be manipulated to produce films. Indeed, gels obtained with HPC concentrations above 6% were too thick for optimal spreading and caused jolts while spreading the gel, resulting in noticeable irregularities in the casting. Conversely, a percentage of HPC below 4% resulted in a gel that was too liquid. Gel with an HPC content between 4% and 6% were found to be acceptable by visual and manual inspection with no significant differences. Therefore, the selection of the final HPC content was made based on the disintegration assay.

Glycerol: the concentrations investigated were 0.5%, 1%, 1.5%, 2%, and 3% with 4% HPC. The 2% and 3% glycerol formulations were considered too adhesive for operator handling and were discarded from the study. Other results are shown in Table 1.

The 0.5% formulation was considered the worst, as the films produced were very brittle. The 1% and 1.5% formulations appear to be adequate, with the 1.5% glycerol formulation scoring the highest on all tests except stickiness, but was still considered pleasant to handle and was thus selected.

These various tests resulted in the formulation shown in Table 2.

**Figure 1:**
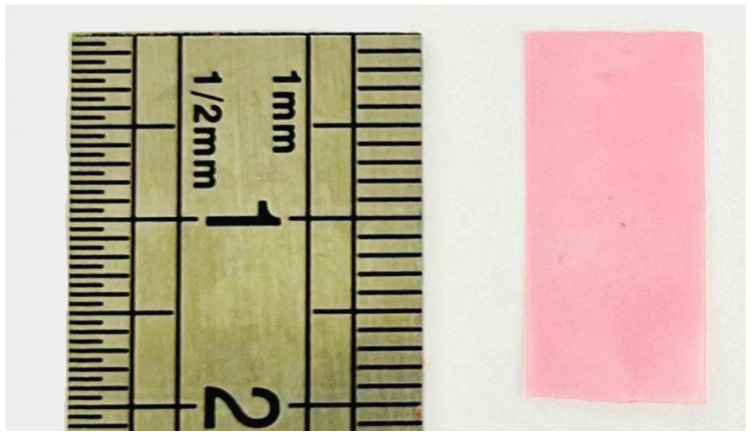
Typical HMS ODF colored with incorporation of carmine red for the purpose of photography.

When this gel formulation was cast with a thickness of 1700 µm, a large film of 0.5 mg/cm^2^ was obtained. After drying, it was cut at 1 × 2 cm to give 1 mg of HCT equivalent per ODF unit. Typical ODFs using our process and formulation are shown in Figure 1.

### 2.3. ODF and Casting Gel Characterization Assay

#### 2.3.1. ODF Disintegration Time

The disintegration times of the ODF prepared with 4%, 5%, or 6% HPC according to the formula detailed in Table 3 were measured and are shown in Table 3.

With 4% HPC, a faster time to the first break as well as a faster time to complete disintegration were obtained. Based on those results, the content of HPC was further set at 4% for the rest of the study.

#### 2.3.2. Casting Gel Content Uniformity

The HMS contents of the upper, middle, and bottom zones were 104.6 ± 1.0%, 103.1 ± 2.0%, and 104.4 ± 1.5% of the target value, respectively. The average content was 104.1 ± 1.5%.

#### 2.3.3. Casting Gel Viscosity

The dynamic viscosity of the degassed gel was measured using an SP-2 probe at 1761 mPa.s^−1^ at 10 rpm and 1686 mPa.s^−1^ at 5 rpm. Using the SP-1 probe, the viscosity was measured at 1830 mPa.s^−1^.

#### 2.3.4. ODF Mass and Thickness Uniformity

The average mass and thickness of dry 2 cm^2^ ODFs after cutting were measured at 26.7 ± 1.7 mg and 0.11 ± 0.01 mm, respectively. ODFs were very similar in each batch at SD < 0.2 mm.

#### 2.3.5. ODF Content Uniformity

The amount of total API (as mean ± SD; n = 3) expressed as HCT equivalent (mg) for 2 cm^2^ ODFs is 1.03 ± 0.06 mg, 0.98 ± 0.11 mg, and 0.96 ± 0.06 mg, in the left, middle, and right areas, respectively. The average content is 98.5% of the target value, and all films were in the range of 100 ± 15% of the expected content.

#### 2.3.6. Casting Gel and Dissolved ODF pH Determination

The pH of the casting gel was measured at 5.5 ± 0.1 (n = 3). The average pH of a water-dissolved HMS ODF was found to be 6.5 ± 0.1 (n = 3).

#### 2.3.7. ODFs Residual Water Content

**Figure 2:**
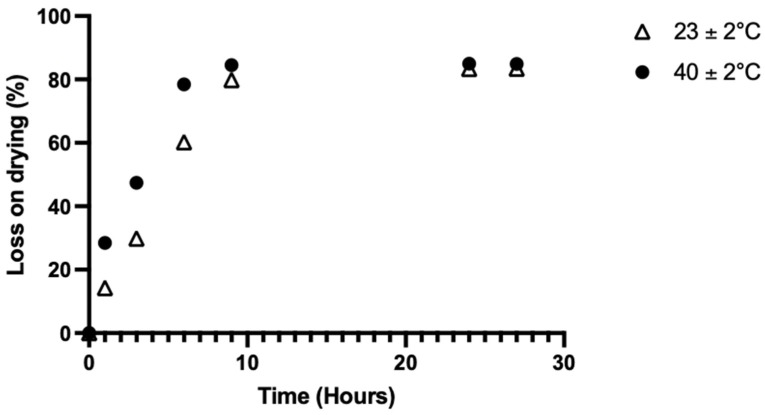
HMS ODF water loss on drying with 4% HPC formula. Standard deviation bars are not visible on the figure due to low data scatter (n = 3).

Drying and solvent evaporation was investigated at three temperatures: room temperature (23 ± 2 °C), 40 ± 2 °C, and 60 ± 2 °C. The results are shown in Figure 2.

The 60 °C condition could not be studied because the gel dephased. After 24 h, the loss from drying was not statistically significant: between 23 ± 2 °C (83.4 ± 0.4 %) and 40 ± 2 °C (84.9 ± 0.1 %). Drying at 40 °C allowed us to reach 78.4 ± 1.3% of loss in 6 h; in comparison, at room temperature, the films reached 79.8 ± 1.5% in 9 h.

The residual water content of the HMS ODF was 9.0 ± 1.2% (n = 3), according to Equation (2), with a weight of 23.6 ± 1.2 mg measured after 1 h.

#### 2.3.8. ODF Dissolution Test

**Figure 3:**
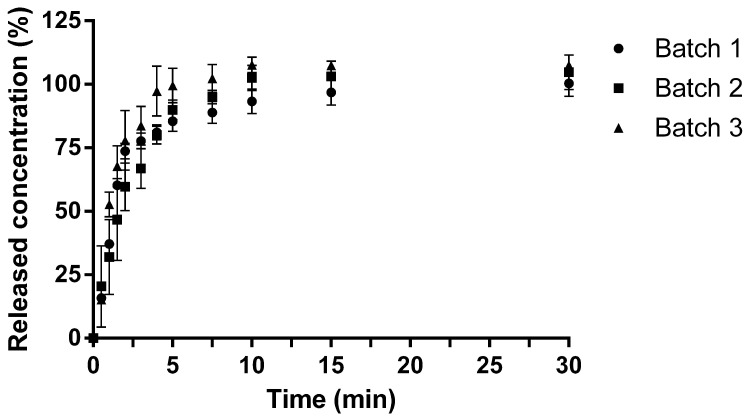
Evolution of HMS and HCT release (%) during ODF dissolution study (mean ± SD; n = 3 per batch; 4% HPC formula).

The results of the dissolution test are shown in Figure 3. Three independent batches were prepared, and three 1 × 2 cm ODFs per batch were tested. The ODFs reached the target concentration of 50.0 ± 2.5 µg/mL after approximately 5 min.

#### 2.3.9. ODFs Stability Study

**Figure 4:**
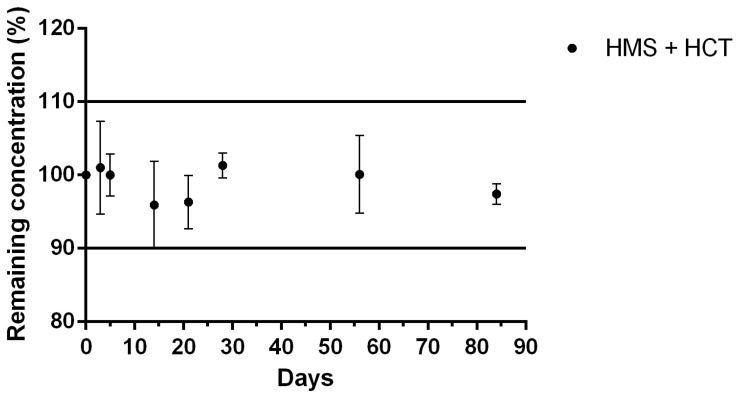
ODF drug content expressed as total HMS and HCT concentration when stored at 23 ± 2 °C, calculated as the ratio of the concentration on the day tested to the concentration on day 0; values are mean ± SD (n = 3; 4% HPC formula).

**Figure 5:**
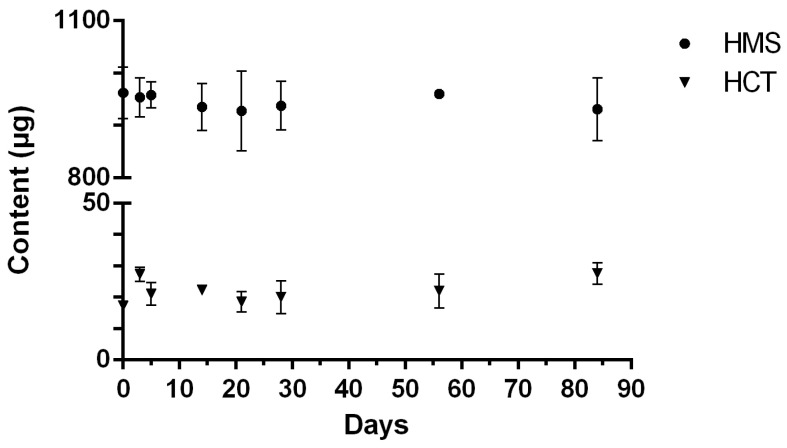
HMS and HCT content in ODFs when stored at 23 ± 2 °C, calculated as the ratio of their concentration to the total concentration (HMS and HCT); values are mean ± SD; n = 3 (4% HPC ODF formula).

No variation of more than 10% in content within a film has been shown (Figure 4). The proportions of HMS and HCT did not otherwise vary during the stability study (Figure 5). In addition, no changes in organoleptic characteristics were noted.

Regarding the unidentified impurity “peak 2”, its relative amount did not change significantly during the stability study (Table S6).

## 3. Discussion

This study demonstrates the feasibility of producing an original ODF formulation containing HMS by solvent-casting in a hospital pharmacy production unit. The excipients in the formulation (i.e., HPC, glycerol, povidone, sucralose, polysorbate 80, and flavoring agent) are commonly used in pharmacies and are readily available. Several preparation methods and formulations were tested during this study. The resulting optimized ODFs were clear to slightly white, thin, and flexible. The viscosity of the casting gel was critical to ensure easy deposition without spreading. This yielded easier and more uniform gel deposition. A variety of HPC casting gels were previously reported in Takeuchi et al. [16]. These authors highlighted an optimum viscosity of the gel between 1000 and 10,000 mPa.s^−1^ for optimum control of the gel thickness during casting. The dynamic viscosity of our 4% HPC gel was measured at ~1760 mPa.s^−1^, which is in line with such recommendations and was found to be adequate for the casting preparation method. Glycerol content was set at 1.5%. While the 1% glycerol formulation showed lower tackiness, the organoleptic touch and the overall ODF handling was better with 1.5%, so the glycerol content was set at 1.5%. If less stickiness were required (e.g., unpacking ODF), reducing the glycerol to 1% could be further considered. Our surfactant level seemed to be well optimized, as the film did not shrink during drying and the dried ODF retained adequate mechanical properties.

According to the European Pharmacopeia, dissolution assay is the only mandatory specific test for ODFs [9]. Complete ODF dissolution was achieved in approximately 5 min, which is in line with European Pharmacopoeia standards requiring a maximum dissolution time of 45 min for a standard release dosage form. Additionally, the uniformity of the mass and thickness of the batches tested were found to be acceptable for production. The different values measured (e.g., mass, thickness, content) were reproducible within the same batch and between different batches. Our preparation method therefore seems adequate and reproducible and is in line with the European Pharmacopoeia standards.

Regarding ODF disintegration, the 4% HPC formulation was found to be more suitable, allowing for a faster disintegration time, and tended to be close to reported ODF disintegration time, which was around 200 to 400 s [17,18]. A lower concentration of 4% HPC has a higher risk of making ODF textures more brittle and fragile. The strength of the ODF can influence the handling of the film and affect the different stages of the film process: handling during production (i.e., demolding, cutting, and packaging), transport, storage, and the unpacking and administration of the ODFs [15]. Increasing the HPC content may be considered if the tensile strength is unsatisfactory. However, this will also lead to a delay in the disintegration time of the ODFs. Although our experiments have shown that the concentration of povidone K25 reached its maximum, the reduction of the disintegration time could also be possibly adjusted by the addition of starch to the ODFs, an excipient that is known for its properties as a superdisintegrant. Although not mandated by the European Pharmacopoeia, the tests we have carried out provides supporting evaluations and are useful for quality control and stability studies of the ODF mechanical properties.

Another critical issue in ODF formulation is the homogeneity of the API content, mainly assessed by mass and content uniformity tests. Previous studies have highlighted the importance of achieving complete API solubilization/dispersion in the aqueous medium to be able to produce an ODF with the required mechanical properties and dosage uniformity [10]. As expected, the use of poorly water-soluble HCT did not achieve these goals. Interestingly, recent studies have investigated different strategies to formulate poorly water-soluble API in lipid dispersions (i.e., nanosupension, nanoemulsion) and integrating them into the ODF matrix [19]. Other API solubilization strategies, such as the use of cyclodextrins, may also be considered. However, the availability of water-soluble hydrocortisone ester of succinate for intravenous use or HMS has enabled its inclusion as the API in our ODF formulations. HMS contains a labile ester function with hydrolysis occurring by spontaneous reaction, including at neutral pH, and is catalyzed under acid or alkaline conditions to rapidly and completely generate both HCT and succinate [20].

Pharmacokinetics in humans following ODF administration have shown that absorption patterns (e.g., Tmax) reflect more an absorption localized in the lower gastro-intestinal tract rather than the oral cavity [21]. The hydrolysis of HMS and the formation of HCT should be readily favored by the different environments and pH conditions found in the gastrointestinal tract. HCT is known to have an oral bioavailability of over 90% [22]. In addition, it has been reported that the plasma elimination half-life of HMS following intravenous dosing is only about 5–6 min, with a peak plasma level of HCT being reached at about 10 min in humans, reinforcing the chemical instability of HMS in vivo [23]. On the basis of these arguments, our hypothesis is that HMS will also be a pro-drug for oral treatment. Pharmacokinetic studies will be important for a better characterization of the HMS ODFs’ oral absorption properties.

A preliminary physicochemical HMS stability study was carried out. ODFs stored at 4 ± 2 °C were brittle after cooling, so only room-temperature (23 ± 2 °C) material was tested. The physicochemical stability was evaluated over a period of 84 days and showed that the HMS content of the ODF stored at 23 ± 2 °C was stable. The proportion of HCT in the ODF did not vary over time and remained stable (~3%). In addition, no changes were observed in the organoleptic characteristics or texture of the ODF. The chromatographic peak impurity (known as peak 2) was found immediately after HMS dissolution, and its content did not change during the entire stability study.

It is interesting to note that ODFs’ low level of residual water can result in better chemical and microbial stability [24]. Indeed, the stability of HMS in solution (equivalent to 1 mg/mL HCT) at pH 5.5, 6.5, or 7.4 and stored at 21 °C was reported to be 4 days only [25], suggesting the greater chemical stability of HMS in the ODF. However, microbiological stability needs to be assessed. Glycerol, also a known preservative, is the only excipient used with reports of toxicity, but the amount used is well below the maximum recommended dose of 10 g [26]. Preservatives such as phenols, parabens, and phtalates are also known to cause adverse effects in growing and pubertal populations [27], while sodium benzoate may cause neonatal neurotoxicity and/or metabolic acidosis [20]. Since ODFs are devoid of such preservatives, they are safer to use than many oral liquid formulations for infants.

ODF manufacturing conditions, and in particular the environment, are critical in controlling microbial contamination. It is essential to limit the contamination of the gel by particles or dust, especially when making and drying the gel. The production of the gel must take place in a controlled environment, ideally in a laminar flow hood, and the drying of the cast gel must take place in a ventilated oven with a HEPA filter. In addition, while dissolution or homogeneous dispersion of the API is essential to ensure the uniformity of the ODF unit produced, the manufacturing process plays a critical role. Indeed, the method described in this study, where a large film is produced and then manually cut into 1 × 2 cm ODF units using a scalpel, is a major source of variability. A more consistent method could be considered, such as the use of pre-formed molds or 3D printing.

The acceptability of children’s medication is crucial because of the characteristics that are inherent to children, in particular difficulty in swallowing and high sensitivity regarding taste [28,29,30]. Recent studies have been conducted comparing palatability and acceptability between ODFs and syrup in pediatric subjects. One study showed improved acceptability in patients between two days and twelve months old and better swallowability in patients over six months of age when compared with syrup [8]. In another report, children aged six months to five years old, including their caregivers, reported good acceptance of ODFs [7]. While the pH value of the HMS ODF is about 6.5, which is consistent with mucosal and oral acceptability, further palatability studies are needed to establish the acceptability of the HMS ODF.

## 4. Materials and Methods

### 4.1. Drugs and Chemicals

Pure micronized pharmaceutical-grade powdered hydrocortisone (HCT), GF-grade hydroxypropylcellulose (HPC) (Klucel GF^®^ 300 mPa.s), sucralose, and povidone K25 were purchased from Inresa Pharmaceutical (Bartheim, France). Hydrocortisone 21-hemisuccinate sodium salt (HMS) was obtained from SERB Pharmaceutical (Hydrocortisone Upjohn^®^; Paris, France). Ondansetron ODF at 4 mg (Setofilm^®^) was purchased from Norgine (Rueil-Malmaison, France). Pharmaceutical-grade polysorbate 80 (tween 80) and glycerol were obtained from Cooper (Melun, France). Pharmaceutical-grade glyceryl monolinoleate (Maisine^®^) was kindly provided by Gattefosse (Saint-Priest, France), and cola flavor was obtained from IFF (Tillburg, The Netherlands). Acetonitrile and orthophosphoric acid were of analytical grade and purchased from VWR chemicals (Fontenay-sous-Bois, France). Distilled sterile water was purchased from Aguettant (Lyon, France).

### 4.2. Validation of Stability-Indicating Liquid Chromatography HMS and HCT Assay

#### 4.2.1. Equipment and Analytical Conditions

The HMS and HCT assays were performed with a high-performance liquid chromatography (HPLC) system (Dionex, Ultimate 3000, Thermo Scientific, Villebon-sur-Yvette, France) with an HPG-3200SD quaternary pump and WPS-3000TSL autosampler, coupled to a Dionex MWD-3000 diode array detector (DAD). HPLC system data acquisition (e.g., peak time, area under peak) was carried out using Chromeleon^®^ software (v6.80 SP2, Thermo Scientific). A Polaris^®^ C18 column (250 × 4.6 mm; particle size, 5 µm; Agilent, Les Ulis, France) was used. The mobile phase consisted of a 30/70% (*v*/*v*) mix of acetonitrile and water adjusted to pH 2.0 with orthophosphoric acid. The flow rate, the injection volume, and the wavelength for quantification were set at 1.0 mL/min, 50 µL, and 254 nm, respectively.

#### 4.2.2. Validation of the HPLC Assay Method

The HMS solution, which was reconstituted and analyzed immediately, was found to contain HCT. Therefore, two analytical methods were developed for HMS and HCT.

The HPLC stability indicating method was developed for the detection and quantification of both HMS and HCT. Their respective calibration curves were used for quantification and validated according to the ICH Q2 guidelines by assessing their linearity, accuracy, specificity, and precision [31].

Two stock solutions of HMS were prepared daily over three days by dilution of HMS with the mobile phase to a final concentration of 1 mg/mL. These stock solutions were used to independently prepare five calibration standards (ranging from 20 and 80 µg/mL) and three quality controls (25, 50, and 75 µg/mL, recorded in triplicate).

Two stock solutions of HCT were also prepared daily over three days by dilution of HCT with the mobile phase to a final concentration of 50 µg/mL. These stock solutions were used for the independent preparation of nine calibration standards (ranging from 0.50 and 50 µg/mL) and three quality controls at each of the following concentrations: 0.8, 5 and 40 µg/mL.

For both calibration curves, the slope, intercept, and correlation coefficient (r^2^) were calculated each day to study linearity. Accuracy was determined using quality controls and expressed as the percentage of recovery determined by the following Equation (1):

(1) $$\frac{\mathrm{Experimental}\;\mathrm{concentration}\;}{\mathrm{Theoretical}\;\mathrm{concentration}}\times \;100$$

with ±5% as acceptance criteria.

The matrix effect was assessed by comparing the calibration curve of HMS or HCT in water and in the presence of all formula excipients.

Method precision was studied using quality controls recorded in triplicate on three independent days. The intra-day analysis was performed by calculating the relative standard deviation (RSD) of the calculated compared to the theoretical concentration of each quality control, recorded in triplicate the same day (n = 3). The inter-day analysis was performed by calculating the RSD of each quality control recorded over three days (n = 9). Additionally, repeatability was estimated by recording 10 times a 50 µg/mL solution of HMS and calculating the RSD.

The limit of detection (LOD) and limit of quantification (LOQ) for HMS and HCT were evaluated based on response standard deviation and calibration curve slope based on the following equation:

LOD = sinterceptsslope×3.3; LOQ = sinterceptsslope, where s(intercept) and s(slope) are the standard deviation of the y-intercept and slope of the calibration curve, respectively.

#### 4.2.3. Selectivity of the HMS and HCT Analytical Method

Solutions of HCT and HMS at 50 µg/mL were prepared to obtain typical chromatograms. The selectivity of the method was ensured so that no chromatographic peak of an excipient or degradation product coincided with that for HMS and HCT. The chromatograms of HMS in the presence of all formula excipients were visually inspected to detect changes in shape of the peak as well as by the analysis of the HMS peak UV spectra recorded by DAD (200–400 nm).

To ensure that the method could be used to assess the stability of active ingredients, forced degradations of HMS and HCT solutions were conducted under three conditions: acidic (0.1 M HCl), alkaline (0.1 M NaOH), and oxidative (3% H_2_O_2_). The temperature was at 23 ± 2 °C for HMS, 40 ± 2 °C for HCT acidic and alkaline stress, and 40 ± 2 °C for both HMS and HCT for the oxidative stress studies. Samples were taken and analyzed at selected time points.

To perform the forced degradation study, two stock solutions of HMS and HCT were prepared (200 µg/mL) according to the GERPAC protocol [32]. These solutions were then diluted in equal parts with either aqueous 0.1 M HCl, 0.1 M NaOH, or 3% H_2_O_2_ and at a controlled temperature. After a selected reaction time, each solution was pH-neutralized if needed, and a mobile phase was added to obtain a final 1/4th dilution.

Chromatograms were visually inspected as well as the HMS and HCT peak UV spectra to detect any changes or coelution of degradation product.

### 4.3. ODF Formulation Studies

#### 4.3.1. Optimizing the Preparation of the Casting Solution and ODFs

ODF were prepared in four main steps: 1. dissolution and mixing excipients and API with water; 2. gel formation and removal of entrapped air; 3. casting of the gel onto a large surface; 4. water evaporation and cutting to the desired size using a ruler and a scalpel, and therefore API dose, into single ODFs.

Each step has been the subject of optimization though the variations of different parameters:

(1) The homogeneity of the casting solution has been improved through formulation testing by comparing the dissolution/content of HCT or the HMS in the casting solution. The order of inclusion of the components and management of the solution temperature (23 °C or 40 °C) were also investigated and evaluated by visual inspection.
(2) Air bubbles were removed with or without five minutes’ sonication (Branson 2510, VWR, Rosny, France) and then left at +4 °C or 23 °C for 10 h and evaluated by visual inspection.
(3) The gel was cast on the casting surface on an area of 5 cm × 20 cm and at a thickness of 1700 µm. The casting surface was evaluated on four different materials: food-grade greaseproof paper, glass, polyolefin liner, and food-grade silicone. Drying was carried out at 23 ± 2 °C for 24 h. This study was performed three times on each surface material.
(4) Drying was assessed by casting 1000 mg of gel onto a food-grade silicone surface and evaluated under three temperature conditions: 23 ± 2 °C, 40 ± 2 °C, and 60 ± 2 °C in the proofer (n = 3 per condition). The weight was measured at different time points using an analytical balance (Mettler Toledo AG204, Viroflay, France) and compared to the initial value for calculation of the water loss on drying (%) using this formula, Equation (2):

(2) $$\mathrm{Loss}\;\mathrm{on}\;\mathrm{drying}=\frac{\mathrm{Weight}\;1-\mathrm{Weight}\;2\;}{\mathrm{Weight}\;1}\;\times \;100,$$

with Weight 1 = the weight of the film at start time (t0), and Weight 2 = the weight of the film at another chosen time.

#### 4.3.2. Formula Optimization

To find an optimal ODF formulation, the selected component proportions were varied as described in Table 4.

The formulations were first assessed by manual and visual inspection on the basis of gel fluidity, ease of spreading, and feasibility of the casting process.

API: an appropriate drug amount of HMS was incorporated to produce 1 × 2 cm ODFs containing the equivalent of 1 mg HCT. The molar mass of HMS being 484.51 g/mol, compared to 362.46 g/mol for HCT, 1 mg of HCT is equivalent to 1.34 mg of HMS.

HPC and povidone: gel viscosity and film hardness were improved by varying the percentages of HPC and povidone. The gel viscosity had to be low enough to allow for smooth casting but not too low to keep the gel fairly compact during drying without spreading. The films produced had to be strong enough not to break during demolding and handling.

Glycerol: the concentration was optimized by the preparation of five batches of films with increasing glycerol concentrations: 0.5%, 1%, 1.5%, 2%, and 3%. Five pharmacists were asked to rate each film on three criteria: flexibility, stretch resistance, and non-stickiness of the film. Each criterion was scored from 1 to 5, with 5 being the highest.

Surfactant: as described by Cuppone et al. [24], the use of a silicone surface requires a surfactant with a hydrophilic/lipophilic balance (HLB) around 4.5 to prevent gel shrinkage during drying. Therefore, a mixture of glyceryl monolinoleate/polysorbate 80 (75/25%; *w*/*w*) with a calculated HLB of 4.5 was chosen.

Sucralose and flavor: cola flavor is described as good at masking bitterness. To this end, it was incorporated with sucralose at the usual fixed concentrations.

### 4.4. ODF Characterization Assay

Once a formulation had been judged satisfactory based on manual handling and visual inspection, the resulting gel and ODFs were evaluated through physicochemical testing.

#### 4.4.1. ODF Disintegration Time

In the absence of a specific European Pharmacopoeia monograph, disintegration was performed using the method described by El-Setouhy et al. [33]. A 1 × 2 cm film (2 cm^2^; 1 mg of HCT) was placed in a Petri dish (diameter: 4.5 cm; VWR) filled with 10 mL of phosphate buffer (0.1 M, pH 6.8). The Petri dish was placed on a hotplate with magnetic stirring (AM3003 Bioblock, Fisher scientific, Illkirch, France). The temperature was maintained at 37 ± 2 °C, and the stirring speed was set at 100 rpm. The time of the first break and the time of complete disintegration were measured using a timer. The first break time was determined visually as the time at which the first film change/alteration occurred. The time at which the observer estimated that almost 90% of the total film had disintegrated was defined as the time of disintegration. Each test was performed in triplicate by the same observer. The disintegration times for the commercial ondansetron ODF, Setofilm^®^ 4 mg, were also measured for comparison.

#### 4.4.2. Drug Content Uniformity of the Casting Gel

The uniformity of the gel’s drug content was assessed by preparing 50 mL of gel and sampling the top, middle, and bottom of the gel. Five samples were taken from each level and analyzed by HPLC for HMS and HCT content.

#### 4.4.3. Casting Gel Viscosity

The dynamic viscosity of the 4% HPC gel, once degassed, was measured using a Rotavisc^®^ Lo-vi (IKA, Staufen Im Breisgau, Deutschland). Measurements were conducted at 23 ± 2 °C for at least 45 min using an SP-2 probe (IKA) at 5 and 10 rpm and an SP-1 probe (IKA) at 3 rpm.

#### 4.4.4. ODF Mass and Thickness Uniformity

The mass uniformity of the single-dose preparations was determined according to the European Pharmacopeia (method 2.9.5) [9]. Twenty ODFs (1 × 2 cm) randomly selected from the same batch were then individually weighed using an analytical balance.

The thickness of twenty ODFs (1 × 2 cm) randomly selected from the same batch was measured using a numeric caliper (Dexter, Lille, France) with a lower limit of detection of 0.1 mm and precision of 0.02 mm.

#### 4.4.5. ODF Content Uniformity

Content uniformity was assessed after casting on a 24.4 × 8.4 cm surface with a thickness of 1700 µm. Once dried, a strip 0.2 cm in width was cut from the end of each side. The film was then divided into three equal sections: left, middle, and right. Each section was then cut into 32 film sections of 1 × 2 cm (corresponding to 1 mg of hydrocortisone base). Three film sections from each area were analyzed for HMS and HCT content by HPLC.

#### 4.4.6. Casting Gel and Dissolved ODF pH Determination

The pH of the casting gel and a dissolved ODF after dissolving a film in 2 mL of distilled water were measured using a pH meter (Mettler-Toledo, Viroflay, France).

#### 4.4.7. ODF Residual Water Content

The measurement of the residual water content was carried out by placing three ODFs in a drying oven (VWR) set at 105 °C for a period of 1 h. The initial and final weights were measured on an analytical balance (Mettler Toledo, Viroflay, France), and water loss was calculated according to Equation (2) [24].

#### 4.4.8. ODF Dissolution Assay

The dissolution assay was performed under sink conditions on three films of 1 × 2 cm and three different batches (n = 9). Each film was placed in a 20 mL vial containing 20 mL of phosphate buffer (0.1 M, pH 6.8). The vials were placed on a magnetic stirrer with a heating plate maintained at 37 °C and a stirring speed of 500 rpm (AM3003 Bioblock, Fisher scientific, Illkirch, France). Twelve samples of 1 mL each were collected at 0, 0.5, 1, 1.5, 2, 3, 4, 5, 7.5, 10, 15, and 30 min using a pipet (Mettler Toledo). Each 1 mL of sample was compensated for with an equal volume of buffer. At each sampling time (n), the amount of HMS and HCT collected at the sampling time (n−1) was added to the measured concentration to compensate for the amount previously sampled. Results are reported as a percentage of drug released (both HMS and HCT) compared to the theoretical concentration corrected for the surface area of the ODF.

#### 4.4.9. ODF Drug Stability Study

ODFs (1 × 2 cm) were placed in Medi-Dose^®^ anti-UV individual blisters (Pero’s, Saint-Priest, France) and stored at ambient temperature (23 ± 2 °C; 55 ± 5% relative humidity). Three ODFs were tested at each selected time point: 0, 1, 2, 3, 5, 7, 10, and 14 days and weekly up to 84 days.

HMS and HCT content were assessed simultaneously using the stability-indicating liquid chromatography and respective calibration curves. Stability was determined by evaluating the percentage of the initial concentration remaining at each time point. Stability is defined as the recovery of at least 90% of the initial combined HMS and HCT concentration. The results are expressed as a percentage of the remaining HMS and HCT content compared to the initial concentration after correction by the surface of the ODF.

Organoleptic parameters such as aspects and texture were also noted at each time point.

### 4.5. Data Analysis

Data analysis was performed using Excel 365 (Microsoft, Seattle, WA, USA) and Prism (GraphPad Software, version 7.04, San Diego, CA, USA). Descriptive statistics for continuous variables were expressed as mean ± standard deviation (SD) unless otherwise specified. One-way ANOVA with appropriate post-test for multiple comparisons was used for statistical analysis (Graph Pad Prism V7.04). The level of statistical significance was set at a *p*-value of less than 0.05.

## 5. Conclusions

In conclusion, the development of ODFs containing HMS as a hydrocortisone prodrug presents a promising alternative to existing formulations for pediatric patients. The ODF formulation demonstrated a long stability, uniformity of content, and rapid disintegration. The method for preparing ODFs was straightforward and cost-effective, making it suitable for production in hospital pharmacies. Additional studies are required to assess the clinical efficacy of this formulation in real-world settings.

## Figures and Tables

**Table 1:** Scoring of the ODF formulations according to the glycerol content (4% HPC formula).

Glycerol	0.5%	1.0%	1.5%
Flexibility (/5) (mean ± SD)	1.4 ± 0.5	3.2 ± 1.1	4.6 ± 0.5
Stretch resistance (/5) (mean ± SD)	1.4 ± 0.9	3.8 ± 1.3	4.2 ± 0.8
Stickiness (/5) (mean ± SD)	4.0 ± 0.7	3.6 ± 0.5	3.0 ± 1.0
Total score (/15) (mean ± SD)	7.2 ±1.5	10.6 ± 0.3	11.8 ± 0.3

SD: Standard deviation.

**Table 2:** Composition of casting gel for HMS ODF.

Ingredients	Composition (% *w*/*v*)
HMS	0.30 (in equivalent HCT)
HPC	4.0–6.0
Glycerol	1.5
Povidone K25	5.0
Surfactant	0.20
Sucralose	0.25
Cola flavor	0.50
Water	Up to 100

**Table 3:** Setofilm^®^ and HMS ODF time for disintegration and breakage (n = 3).

	*Setofilm^®^*	*HPC 4%*	*HPC 5%*	*HPC 6%*
*First break time (mean ± SD) (s)*	35 ± 10	62 ± 39	264 ± 68 *	511 ± 101 **
*Time for complete disintegration (mean ± SD) (s)*	219 ± 149	541 ± 86 *	1 620 ± 114 ***	2 073 ± 234 ***

SD: Standard deviation; (* *p* < 0.05; ** *p* < 0.01; *** *p* < 0.001 as compared to Setofilm^®^).

**Table 4:** Concentration range of excipients tested for optimization of the ODF formulation.

Ingredients	Range Tested (% *w*/*v*)
HMS	0.30 (in equivalent HCT)
HPC	2–6
Glycerol	0.5–3.0
Povidone K25	5–10
Surfactant	0–0.4
Sucralose	0.25
Cola flavor	0.50
Water	Up to 100

HMS: Hydrocortisone 21-hemisuccinate sodium salt; HCT: hydrocortisone; HPC: hydroxypropylcellulose.

## Data Availability

All main data are available in the manuscript and Supplementary Materials.

## Supplementary Materials

The following supporting information can be downloaded at: https://www.mdpi.com/article/10.3390/ph18010086/s1, Figure S1: (A) Typical chromatogram of HMS. Insert pointed by an arrow: UV spectra of HMS with x axis the wavelength (200–330 nm), and absorbance response in y axis; (B) Typical chromatogram of pure HCT. Insert pointed by an arrow: UV spectra of HCT measured between 200–330 nm (x axis) and absorbance response (y axis); Figure S2: (A): Comparison of chromatogram of forced degradation of HCT. HCT control (black); acidic condition (blue); alkaline condition (pink); HMS control (crimson red). (B) Comparison of chromatograms of forced degradation of HMS. HMS control (black); alkaline condition (blue); acidic condition (pink); Figure S3: Chromatogram and 3D UV-plot (200–400 nm) of HMS after forced degradation in (A) HCl 0.1 M; 23 °C; 15 min (B) NaOH 0.1 M; 23 °C; 15 min (C) H_2_O_2_ 3%; 40 °C; 24 h; Table S1: Precision and accuracy of the HMS calibration curve; Table S2: Intra and inter-day precision and accuracy results at three HMS concentration levels; Table S3: Precision and accuracy of the HCT calibration curve; Table S4: Intra and inter-day precision and accuracy results at three HCT concentration levels; Table S5: Forced degradation of the HCT and HMS; Table S6: Chromatographic area ratio of peak 2 to HMS during the stability study.
