# Supplementary material for: Development of a Hydrocortisone Orodispersible Thin Film Containing Its Succinate Prodrug
Source: Pharmaceuticals (Basel). 2025 Jan 13;18(1):86. doi: 10.3390/ph18010086 (PMC11768698; doi:10.3390/ph18010086)
Supplement: Supplementary file 1 [file pharmaceuticals-18-00086-s001.zip › pharmaceuticals-3355325-supplementary.pdf]

pharmaceuticals

MDPI

Supplemental data

Development of a Hydrocortisone Orodispersible Thin Film Containing Its Succinate Prodrug

Supplemental Figure

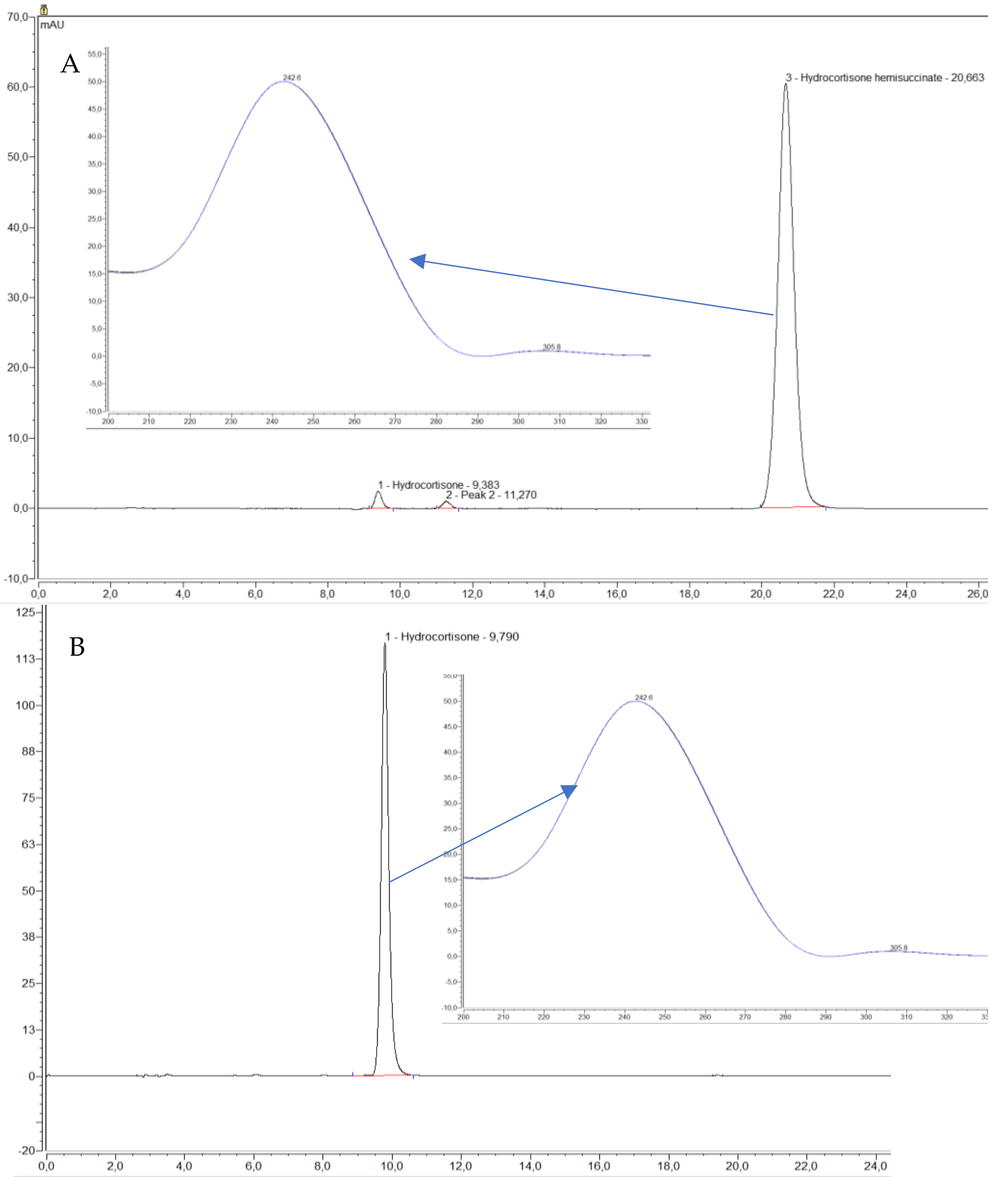

Figure S1. (A) Typical chromatogram of HMS . Insert pointed by an arrow: UV spectra of HMS with x axis the wavelength (200-330 nm), and absorbance response in y axis; (B) Typical chromatogram of pure HCT. Insert pointed by an arrow: UV spectra of HCT measured between 200-330nm (x axis) and absorbance response (y axis)

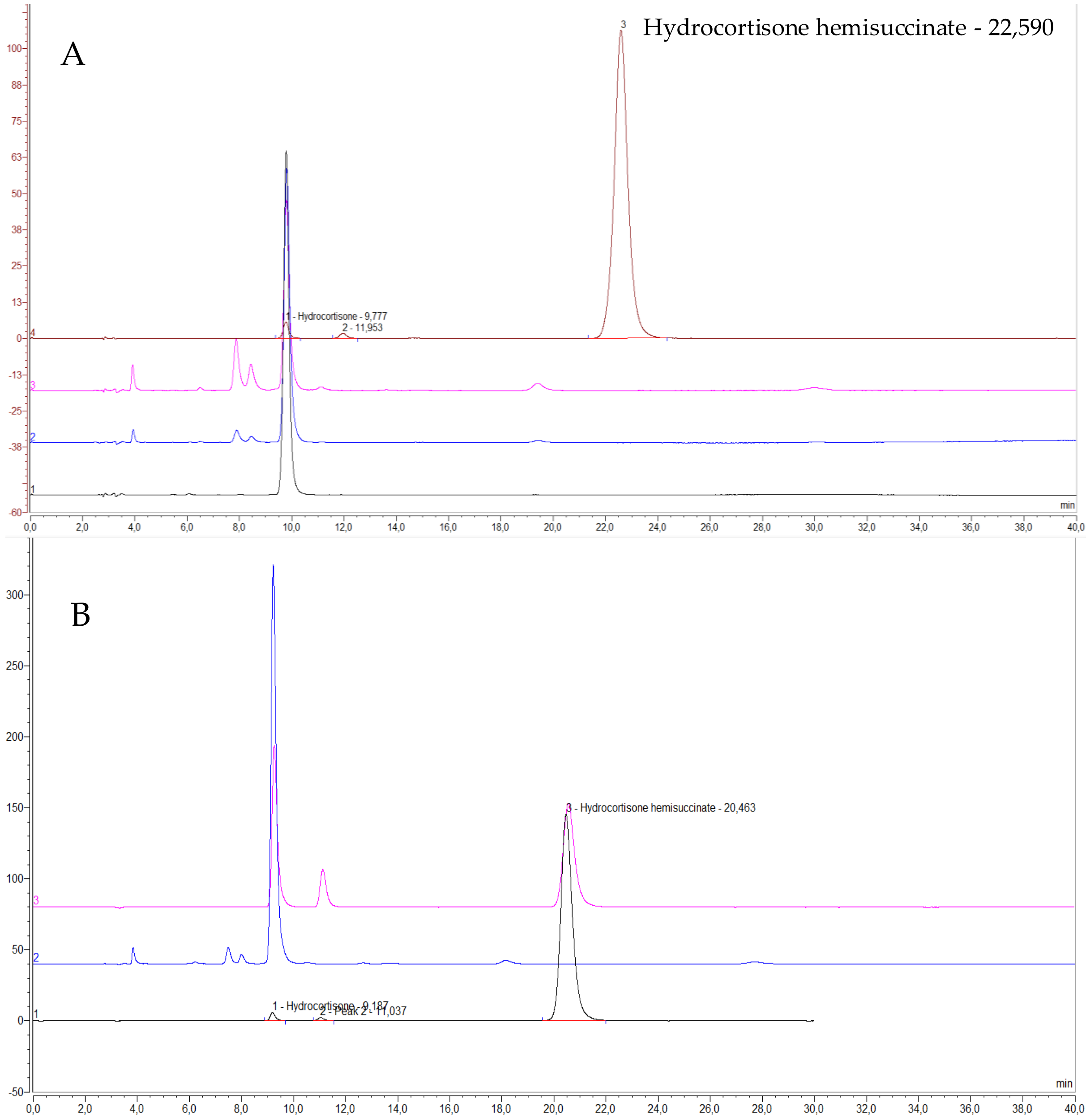

Figure S2. (A): Comparison of chromatogram of forced degradation of HCT. HCT control (black); acidic condition (Blue); alkaline condition (pink); HMS control (crimson red). (B) Comparison of chromatograms of forced degradation of HMS. HMS control (Black); alkaline condition (blue); acidic condition (pink)

(A)

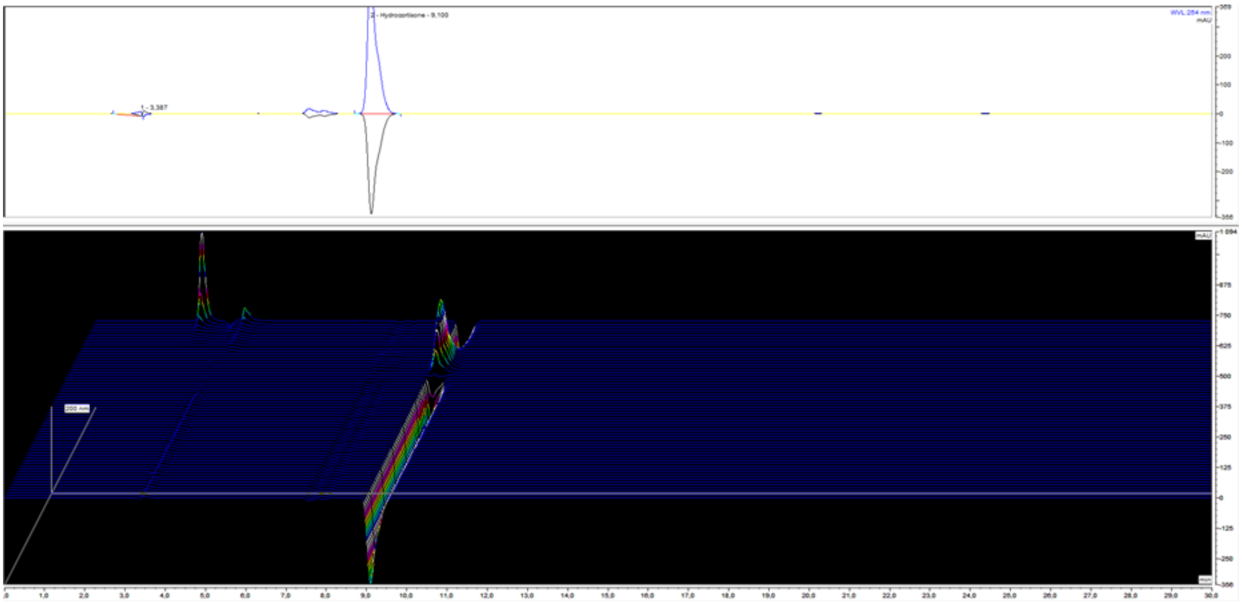

(B)

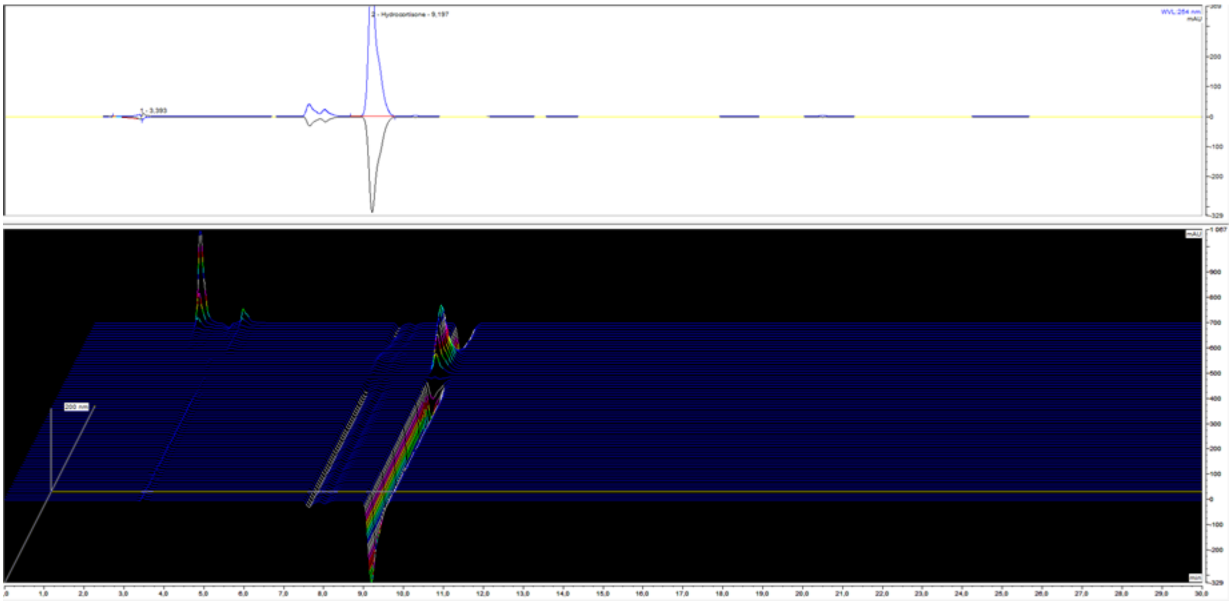

(C)

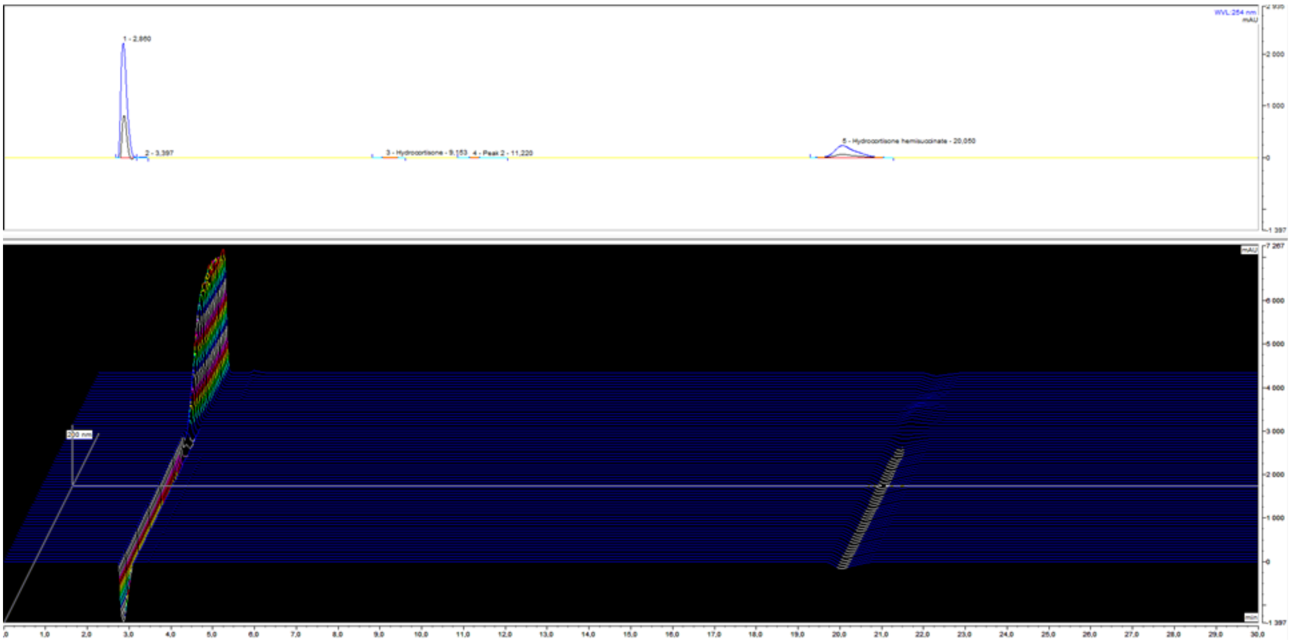

Figure S3. Chromatogram and 3D UV-plot (200-400 nm) of HMS after forced degradation in (A) HCl 0.1M; 23°C; 15min (B) NaOH 0.1M; 23°C; 15min (C) H_2O_2 3% ; 40°C; 24h

Supplemental Table

Table S1. Precision and accuracy of the HMS calibration curve

HMS Concentration (µg/mL)	Calculated concentration ± SD (µg/mL)	RSD (%)	Accuracy (%)
20	19.9± 0.4	1.8%	99.3 ± 1.8
35	35.0 ± 0.8	2.2%	99.9 ± 2.2
50	50.1 ± 1.1	2.1%	100.3 ± 2.2
65	65.4 ± 1.5	2.2%	100.6 ± 2.2
80	79.7 ± 1.9	2.4%	99.6 ± 2.4

SD : Standard deviation ; RSD : Relative standard deviation

Table S2. Intra and inter-day precision and accuracy results at three HMS concentration levels

HMS Theoretical Concentration (µg/mL)	Day	Intra-Day Validation			Inter-Day Validation		
		Mean calculated Concentration ± SD (µg/mL) (*n* = 9)	RSD (%)	Accuracy (%)	Mean calculated Concentration ± SD (µg/mL) (*n* = 9)	RSD (%)	Mean Accuracy ± SD (%)
	1	25.1 ± 0.1	0.5	100.5			
**25**	2	24.9 ± 0.1	0.3	99.6	24.8 ± 0.4	1.6	99.1 ± 1.6
	3	24.3 ± 0.1	0.3	97.4			
	1	51.7 ± 0.1	0.1	103.5			
**50**	2	49.3 ± 0.1	0.1	98.7	49.7 ± 1.8	3.8	99.4 ± 3.8
	3	48 ± 0.1	0.3	96.1			
	1	77.1 ± 0.1	0.2	102.8			
**75**	2	74.5 ± 0.1	0.1	99.3	74.4 ± 2.8	3.8	99.1 ± 3.8
	3	71.4 ± 0.3	0.5	95.3			

SD : Standard deviation ; RSD : Relative standard deviation

Table S3. Precision and accuracy of the HCT calibration curve

HCT Concentration (µg/mL)	Calculated concentration ± SD (µg/mL)	RSD (%)	Accuracy (%)
0,5	0.5 ± 0.1	1.18	95.4 ± 1.1
1	1.0 ± 0.1	1.90	100.5 ± 1.9
4	3.9 ± 0.1	0.06	96.4 ± 0.1
10	10.2 ± 0.1	0.54	101.9 ± 0.5
50	50.0 ± 0.1	0.26	99.9 ± 0.3

SD : Standard deviation ; RSD : Relative standard deviation

Table S4. Intra and inter-day precision and accuracy results at three HCT concentration levels

**HCT Theoretical Concentration (µg/mL)**	Day	**Intra-Day Validation** Mean calculated Concentration ± SD (µg/mL) (*n* = 9)	RSD (%)	Accuracy (%)	**Inter-Day Validation** Mean calculated Concentration ± SD (µg/mL) (*n* = 9)	RSD (%)	Mean Accuracy ± SD (%)
**0.8**	1	0.8 ± 0.1	5.3	103.0	0.8 ± 0.1	1.62%	101.1 ± 1.6
	2	0.8 ± 0.1	2.7	100.5			
	3	0.8 ± 0.1	1.5	99.9			
**5**	1	5.1 ± 0.1	2.6	102.4	5.1 ± 0.1	0.27%	102.4 ± 0.3
	2	5.1 ± 0.1	1.9	102.7			
	3	5.1 ± 0.1	2.7	102.1			
**40**	1	39.4 ± 0.1	0.2	98.6	39.5 ± 0.1	0.24%	98.8 ± 0.2
	2	39.5 ± 0.2	0.5	98.6			
	3	39.6 ± 0.3	0.4	99.0			

SD : Standard deviation ; RSD : Relative standard deviation

Table ***S5*****.** Forced degradation of the HCT and HMS

Drug	Stress Conditions and Time of Analysis	% Remaining	Retention Time of the Detected Degradation Product(s) (min)
**HCT**	Acidic (0.1M, 40°C, 30 min)	77%	3.9; 7.8; 8.4; 19.4
	Alkaline (0.1M, 40°C, 30 min)	53%	3.9; 6.5; 7.8; 8.4; 11.1; 19.4; 30.1
	Oxidative (3%, 40°C, 24h)	90%	No degradation peak detected
	(Heating, 40°C, 24h)	92%	No degradation peak detected
**HMS**	Acidic (0.1M, 23°C, 15 min)	52%	9.2; 11.1
	Alkaline (0.1M, 23°C, 15 min)	< LOQ	7.7; 8.1; 9.2
	Oxidative (3%, 40°C, 24h)	77%	No degradation peak detected
	(Heating, 40°C, 24h)	97%	No degradation peak detected

HCT: hydrocortisone; HMS: hydrocortisone hemisuccinate

Table S6. Chromatographic area ratio of peak 2 to HMS during the stability study. Values are mean (± SD); n=3

	D0	D5	D14	D28	D56	D84
**Average chromatographic area peak ratio (peak 2/HMS)**	1.8% (±0.1%)	2.0% (±0.2%)	2.2% (±0.2%)	2.2% (±0.1%)	2.2% (±0.1%)	2.3% (±0.1%)
